# The energy-converting hydrogenase Ech2 is important for the growth of the thermophilic acetogen *Thermoanaerobacter kivui* on ferredoxin-dependent substrates

**DOI:** 10.1128/spectrum.03380-23

**Published:** 2024-02-22

**Authors:** Christoph Baum, Benjamin Zeldes, Anja Poehlein, Rolf Daniel, Volker Müller, Mirko Basen

**Affiliations:** 1 Microbiology, Institute for Biological Sciences, University of Rostock, Rostock, Germany; 2 Genomic and Applied Microbiology & Göttingen Genomics Laboratory, Georg-August University, Göttingen, Germany; 3 Department of Molecular Microbiology & Bioenergetics, Institute of Molecular Biosciences, Johann Wolfgang Goethe University, Frankfurt am Main, Germany; 4 Department of Maritime Systems, Interdisciplinary Faculty, University of Rostock, Rostock, Germany; Oklahoma State University, Stillwater, Oklahoma, USA

**Keywords:** *Thermoanaerobacter kivui*, thermophilic, acetogen, energy-converting hydrogenase, ferredoxin, carbon monoxide, pyruvate

## Abstract

*Thermoanaerobacter kivui* is the thermophilic acetogenic bacterium with the highest temperature optimum (66°C) and with high growth rates on hydrogen (H_2_) plus carbon dioxide (CO_2_). The bioenergetic model suggests that its redox and energy metabolism depends on energy-converting hydrogenases (Ech). Its genome encodes two Echs, Ech1 and Ech2, as sole coupling sites for energy conservation during growth on H_2_ + CO_2_. During growth on other substrates, its redox activity, the (proton-gradient-coupled) oxidation of H_2_ may be essential to provide reduced ferredoxin (Fd) to the cell. While Ech activity has been demonstrated biochemically, the physiological function of both Ech’s is unclear. Toward that, we deleted the complete gene cluster encoding Ech2. Surprisingly, the *ech2* mutant grew as fast as the wild type on sugar substrates and H_2_ + CO_2_. Hence, Ech1 may be the essential enzyme for energy conservation, and either Ech1 or another enzyme may substitute for H_2_-dependent Fd reduction during growth on sugar substrates, putatively the H_2_-dependent CO_2_ reductase (HDCR). Growth on pyruvate and CO, substrates that are oxidized by Fd-dependent enzymes, was significantly impaired, but to a different extent. While *∆ech2* grew well on pyruvate after four transfers, *∆ech2* did not adapt to CO. Cell suspensions of *∆ech2* converted pyruvate to acetate, but no acetate was produced from CO. We analyzed the genome of five *T. kivui* strains adapted to CO. Strikingly, all strains carried mutations in the hycB3 subunit of HDCR. These mutations are obviously essential for the growth on CO but may inhibit its ability to utilize Fd as substrate.

**IMPORTANCE:**

Acetogens thrive by converting H_2_+CO_2_ to acetate. Under environmental conditions, this allows for only very little energy to be conserved (∆G′<–20 kJ mol^−1^). CO_2_ serves as a terminal electron acceptor in the ancient Wood-Ljungdahl pathway (WLP). Since the WLP is ATP neutral, energy conservation during growth on H_2_ + CO_2_ is dependent on the redox metabolism. Two types of acetogens can be distinguished, Rnf- and Ech-type. The function of both membrane-bound enzyme complexes is twofold—energy conversion and redox balancing. Ech couples the Fd-dependent reduction of protons to H_2_ to the formation of a proton gradient in the thermophilic bacterium *Thermoanaerobacter kivui*. This bacterium may be utilized in gas fermentation at high temperatures, due to very high conversion rates and the availability of genetic tools. The physiological function of an Ech hydrogenase in *T. kivui* was studied to contribute an understanding of its energy and redox metabolism, a prerequisite for future industrial applications.

## INTRODUCTION

Acetogens thrive on the conversion of hydrogen (H_2_) + carbon dioxide (CO_2_) to acetic acid (1). This presents an energetic problem since acetogenesis from H_2_ + CO_2_ at low ambient H_2_ partial pressures (>1 µM) only yields a free energy of about –20 kJ mol^–1^, close to the thermodynamic limit of life (2, 3). Acetogens respire CO_2_, using the reductive Acetyl-Coenzyme A (Acetyl-CoA) pathway or Wood-Ljungdahl pathway (WLP) as the terminal electron accepting pathway (1). Physiologically, they must couple this reduction of CO_2_ to the conservation of energy in the form of a chemiosmotic ion gradient since reductive acetogenesis from H_2_ + CO_2_ primarily does not involve substrate-level phosphorylation. In the WLP, initially, one ATP must be invested to bind formate to tetrahydrofolate, which is reclaimed in the acetate kinase reaction during catabolic conversion of the product of the WLP, acetyl-Coenzyme A (4) to acetate. As a consequence, the WLP in conjunction with the formation of acetate is ATP-neutral, and it has been a long-standing question how autotrophic acetogenesis can be coupled to energy conservation. Beyond acetate kinase, no further reaction allows for substrate-level phosphorylation, and since all enzymes of the pathway are cytoplasmic, none of the reactions can be directly coupled to the build-up of a transmembrane ion gradient (∆µ_i_). Moreover, to facilitate acetate formation, eight reducing equivalents are required to reduce two molecules of CO_2_, and the electron donors for the reductases and dehydrogenases in the WLP differ among species (5–7). During growth on H_2_ + CO_2_, electrons need to be transferred from H_2_ to ferredoxin (Fd), the donor for the CODH, and putatively, to NADPH and/or NADH, which are involved in reducing CO_2_ to formate and methenyl-THF to methyl-THF (3, 5, 8, 9), requiring a complex redox network of different oxidoreductases. The enigma of redox metabolism and energy conservation during growth on H_2_ + CO_2_ have been solved for *Acetobacterium woodii*. In this mesophilic acetogen (T_OPT_ 30°C), H_2_ is oxidized by two hydrogenases. Interestingly, part of the H_2_ is oxidized by a soluble enzyme complex, the hydrogen-dependent carbon dioxide reductase HDCR (10), a soluble enzyme that directly reduces CO_2_ to formate in the first step of the methyl branch of the WLP. The remaining six reducing equivalents needed to reduce formate to a methyl group in the methyl branch of the WLP, and CO_2_ to [CO] in the carbonyl branch are provided by the electron-bifurcating hydrogenase HydABCD (10). However, there is a redox imbalance since the remaining reactions of the WLP need 2 NADH and 1 Fd_red_, and HydABCD provides equal amounts of NADH and Fd_red_. A membrane-bound multisubunit enzyme, the Rnf complex, uses the excess Fd_red_ and reduces NAD^+^, and the free energy of this exergonic reaction is conserved as sodium ion-dependent membrane potential (11, 12). Thus, the complex fulfills both, redox homeostasis and energy conservation (13).

The thermophilic model acetogen *Thermoanaerobacter kivui* (T_OPT_ 66°C) does not possess a Rnf complex. Instead, its genome has *two* gene clusters for energy-converting hydrogenases (Ech), *ech1* (nine genes) and *ech2* (eight genes) as sole membrane-bound putative respiratory enzymes (14). Ech hydrogenases catalyze the reversible oxidation of Fd_red_ with protons as electron acceptors (yielding molecular H_2_) coupled to the build-up of a transmembrane ion gradient in archaea (15–18). Ech from *T. kivui* was demonstrated to carry out this chemiosmotic energy conservation (19), but it was not clear whether Ech1 or Ech2 or both complexes were responsible for the observed activity. The redox and energy metabolism of *T. kivui* is also distinctly different from that of *A. woodii* since many key enzymes have different cofactor specificities (3). The electron-bifurcating hydrogenase HydABC and methylene-THF dehydrogenase are NADPH-dependent (3). Moreover, the organism possesses a transhydrogenase Nfn (NADPH:Fd NADH oxidoreductase). According to the current model, either Ech may therefore occur in two forms, membrane-bound or in a membrane complex with the methylene-THF reductase (MetFV), and depending on the substrate, the complexes are proposed to operate in the direction of Fd oxidation or reduction (3). Since the genetic methods for *T. kivui* (20) allow the overproduction of tagged proteins (7), Ech2 was recently purified and biochemically characterized (21). The enzyme was not found in a super-complex with MetFV and was shown to be dependent on ferredoxin, thereby translocating protons into liposomes (21). The function of Ech2 in redox and energy metabolism, however, has not been elucidated.

Here, we describe the deletion of the complete operon encoding Ech2 in *T. kivui*. The phenotype of the deletion mutant was studied in growth experiments and in resting cells to elucidate the metabolic function of the enzyme complex. Surprisingly, Ech2 was not essential during growth with sugar substrates and on H_2_ + CO_2_. The results are discussed in the context of the current bioenergetic model of *T. kivui*.

## RESULTS AND DISCUSSION

### Generation of an Ech2 deletion mutant

**Fig 1:**
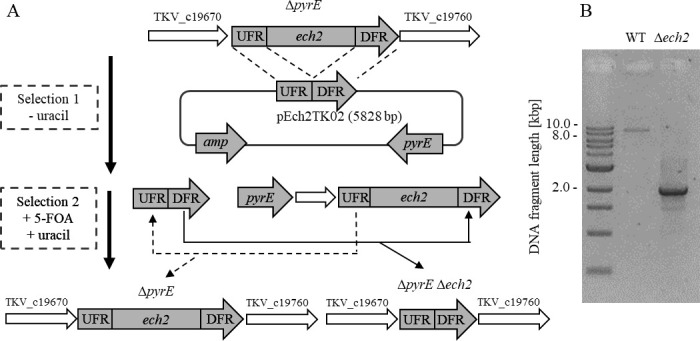
(**A**) Deletion of the entire operon encoding Ech2 *via* two independent homologous recombination events using plasmid pEch2TK02. Plasmid pEch2Tk02 contained homologous regions for integration at the 5′ or 3′ region flanking the *ech2* operon (UFR, upstream flanking region; DFR, downstream flanking region) into the parent strain, *T. kivui* TKV002 (TKV_MB002) lacking *pyrE*; and *ech2* operon replacement *via* homologous recombination. For methodological details, see Material and Methods. (**B**) 1% Agarose gel stained with midori green (Biozym, Hessisch Oldendorf, Germany). The loss of *ech2* (6,770 bp) was verified by PCR using primers binding outside of the *ech2* cluster. Shown is the electrophoretic separation of the DNA fragments from the PCRs using cells of *T. kivui* wild type (WT) (expected fragment size, 8.8 kbp) and ∆*ech2* mutant (expected fragment size, 2.0 kbp).

To resolve the metabolic function of Ech2 in energy and redox metabolism of *T. kivui*, a markerless deletion of the entire 6.77 kb gene cluster encoding the membrane-bound hydrogenase Ech2 was performed using methods for genome manipulations, as described by Basen et al. (20). The uracil-auxotrophic strain *T. kivui* TKV002 (TKV_MB002; ∆*pyrE*) (20) was transformed with the plasmid pE2TK02, which contained *pyrE* and upstream (0.9 kb) and downstream (1.0 kb) flanking regions (UFR and DFR, respectively) of the *ech2* gene cluster (14). Two consecutive steps were performed. First, the plasmid was integrated into the genome, allowing the transformants to grow on minimal media since the plasmid contained *pyrE* (Fig. 1A). In the second step, selection against *pyrE* was carried out using 5-fluoro orotic acid (5-FOA, selection 2). Of the ten colonies grown in the presence of 5-FOA, seven were revertants that had lost the *pyrE* gene, but not the *ech2* cluster, the other three lost the entire *ech2* cluster, as intended (Fig. 1B). Sequencing and qPCR confirmed the genotype of the resulting deletion mutant strain (*T. kivui* ∆*ech2D– ech2F; TKV_c19750– TKV_c19680;* ∆*pyrE*). The strain was designated *T. kivui* TKV_MB050 and will be called ∆*ech2* mutant herein.

### The ∆*ech2* mutant grows on sugar substrates and H_2_+ CO_2_

**Fig 2:**
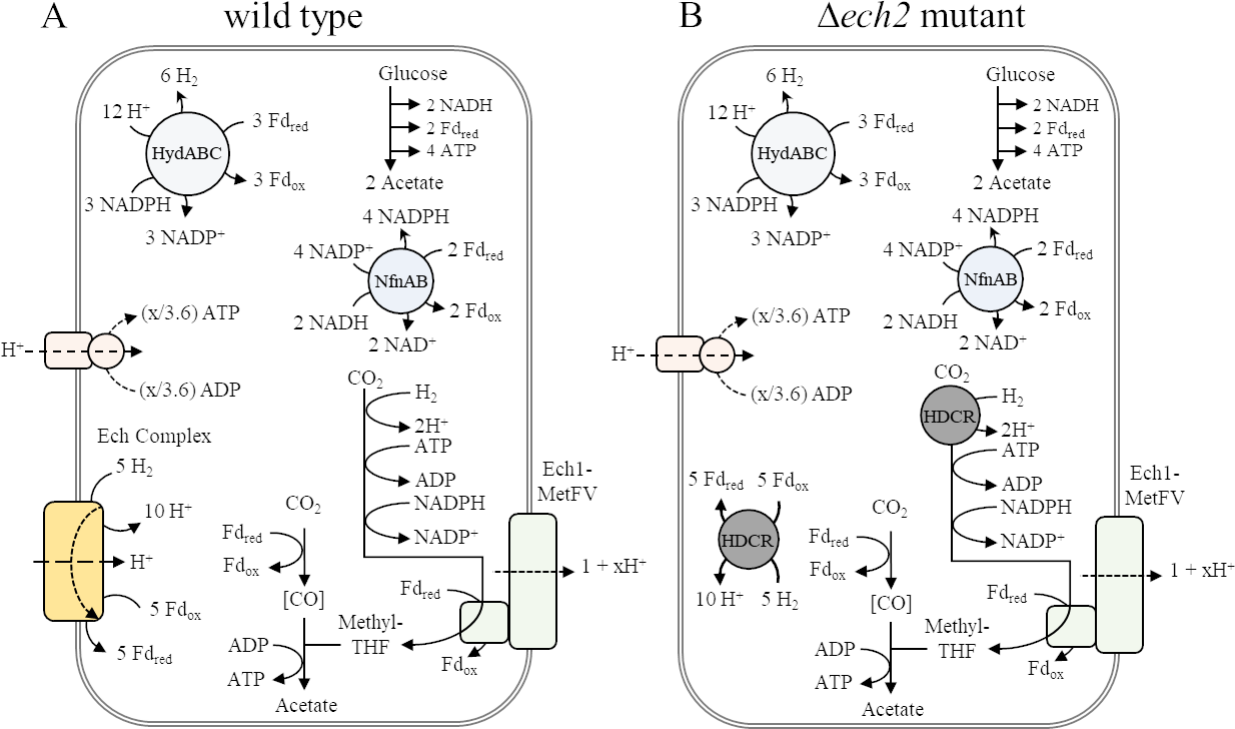
Model of acetogenesis from glucose in *Thermoanaerobacter kivui* (**A**) wild type (3) and (**B**) *ech2* mutant. (**A**) Glucose oxidation provides the required reducing equivalents for the reduction of CO_2_ in the WLP. The electron-bifurcating transhydrogenase Nfn concomitantly oxidizes NADH and Fd_red_ from glucose and pyruvate oxidation to provide NADPH to the WLP and to the electron-bifurcating hydrogenase (HydABC) that oxidizes NADPH and Fd_red_. The produced H_2_ is used by HDCR in the WLP. To balance redox carriers, the energy-conserving hydrogenase (Ech complex) oxidizes H_2_ to reduce ferredoxin (Fd_ox_). (**B**) Hypothetical takeover of Ech2 function by the HDCR. Ech1-MetFV is assumed to translocate 1 + x H^+^ across the membrane. The generated proton motive force is used to synthesize ATP *via* ATP synthase.

Ech hydrogenases are proposed to have different roles during growth with sugar substrates and H_2_ + CO_2_ in *T. kivui*. As membrane-bound enzyme complexes, they couple two fundamental metabolic functions, the oxidation/reduction of redox carriers (Fd_ox_/Fd_red_ and H^+^/H_2_) which is either driven by or generating a transmembrane proton gradient (13). In *T. kivui*, sugars are presumably taken up by PTS systems (22), and the sugar phosphates are then oxidized in glycolysis (yielding NADH) and by pyruvate:ferredoxin oxidoreductase (yielding Fd_red_) to acetate (Fig. 2). The reduced electron carriers NADH and (part of the) Fd_red_ are oxidized by a transhydrogenase NfnAB, producing NADPH, part of which is oxidized by the methylene THF dehydrogenase, and another part is oxidized concomitantly with Fd_red_ by the electron-bifurcating hydrogenase HydABC (3). This leaves internally produced H_2_, part of which is used to activate CO_2_ to formate in the HDCR reaction. *T. kivui* is the only acetogen to date besides *A. woodii* (23) with a characterized HDCR for direct reduction of CO_2_ to formate using electrons from H_2_. The HDCR is extremely active, abundant in the cells (24), and essential for autotrophic growth (25). Recently, HDCR of *T. kivui* has been demonstrated to form bundles of nanowire-like structures attached to one cell pole (24). To balance the redox metabolism on H_2_ + CO_2_ and sugars, H_2_ must be oxidized and Fd_ox_ reduced at the expense of a transmembrane proton gradient, as demonstrated for the purified Ech2 complex (21).

**Fig 3:**
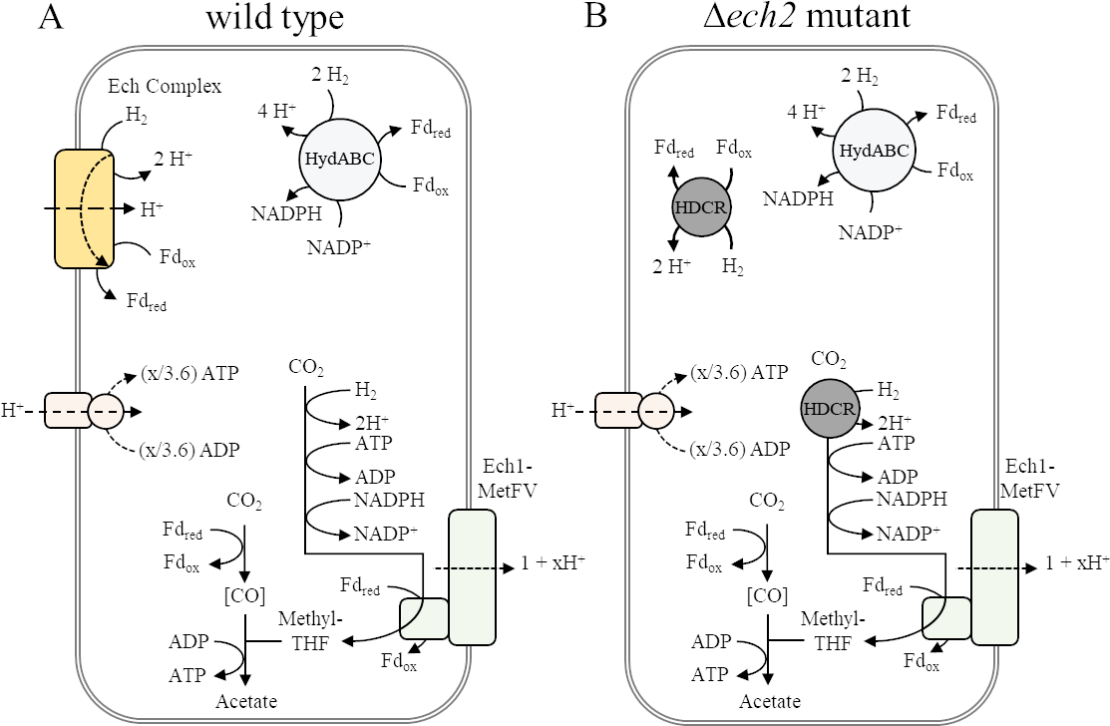
Model of acetogenesis from H_2_ + CO_2_ in *Thermoanaerobacter kivui* (**A**) wild type (3) and (**B**) *ech2* mutant. (**A**) H_2_ oxidation provides the required reducing equivalents for the reduction of CO_2_ in the WLP. The electron-bifurcating hydrogenase (HydABC) and the energy-conserving hydrogenase (Ech complex) oxidize H_2_ to reduce ferredoxin (Fd_ox_) and NADP^+^ to Fd_red_ and NADPH. (**B**) In the *ech2* mutant, HDCR may catalyze the Fd-dependent H_2_ oxidation. Ech1-MetFV is assumed to translocate 1 + x H^+^ across the membrane. The generated proton motive force is used to synthesize ATP *via* ATP synthase.

Since it is unclear whether Ech1 can oxidize Fd_red_ (unpublished observation, V. Müller), we anticipated Ech2 to be essential for Fd_ox_ reduction during growth on sugars as described above, and to be the coupling site for energy conversion during growth on H_2_ + CO_2_ (Fig. 3A). We performed gene expression analysis by RT-qPCR and found that *ech2D* as representative gene of the *ech2* operon was expressed comparable to the housekeeping gene *gyrase* (TKV_c00100, *gyrB*), in cells grown with glucose (1.4-fold) and in cells grown with H_2_+CO_2_ (1.9-fold) (Table 1). *ech1A* was present at a higher level, however, was only slightly upregulated in the presence of H_2_ + CO_2_ (in wild type 7.6-fold vs. *gyrB*, in ∆*ech2* 6.8-fold vs. *gyrB*) compared to glucose (in wild type 4.6- fold vs. *gyrB*, in ∆*ech2* 3.6-fold vs. *gyrB*). This result is in contrast to the previously reported 6-fold and 16-fold higher expression of *ech1* and *ech2*, respectively, with the growth of the wild type on H_2_ + CO_2_, which may be growth phase dependent (19). As expected, in the mutant *ech2D* was not detected.

**TABLE 1:** Expression levels of *ech1*A and *ech2*D compared to the expression level of the housekeeping gene *gyrB* (2^-∆Ct^) in cells of the *T. kivui ech2* mutant and the wild type (DSM2030) as determined by RT- qPCR. The cells were grown in complex medium with either 25 mM glucose or with H_2_/CO_2_ (66/33, vol/vol, 2 atm), and the fourth and seventh rows show the differential expression level (2^-∆∆Ct^)^*a*^

Condition	Wild type	∆*ech2* mutant
*ech1*A vs. *gyrB* (H_2_/CO_2_)	7.6	6.8
*ech1*A vs. *gyrB* (glucose)	4.6	3.6
*ech1*A (H_2_/CO_2_ vs. glucose)	1.7	1.9
*ech2*D vs. *gyrB* (H_2_/CO_2_)	1.9	n.d.
*ech2*D vs. *gyrB* (glucose)	1.4	n.d.
*ech2D* ((H_2_/CO_2_ vs. glucose)	1.4	-

^*a*^ The *ech2D* was not detectable (n.d.) in the ∆*ech2* mutant. Therefore, no differential expression level could be calculated.

**Fig 4:**
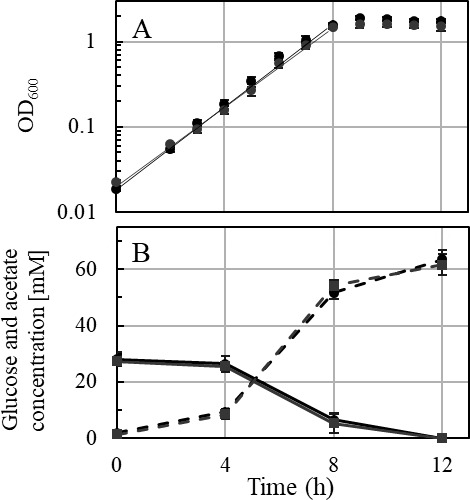
(**A**) Representative growth curve of the *T. kivui* Δ*ech2* mutant (gray) and the wild type, strain DSM 2030 (black) with 25 mM glucose. A representative growth curve is shown. (**B**) Concentrations of glucose (continuous line) and acetate (dashed line). All experiments were performed on complex medium at 65°C and 160 rpm (*n* = 3).

**Fig 5:**
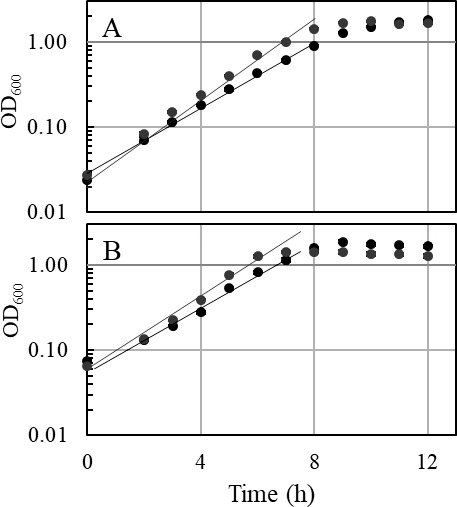
Representative growth curve of the *T. kivui* Δ*ech2* mutant (gray) and the wild type, strain DSM 2030 (black) with (**A**) 25 mM mannitol and (**B**) 25 mM fructose. All experiments were performed on complex medium at 65°C and 160 rpm (*n* = 3).

**Fig 6:**
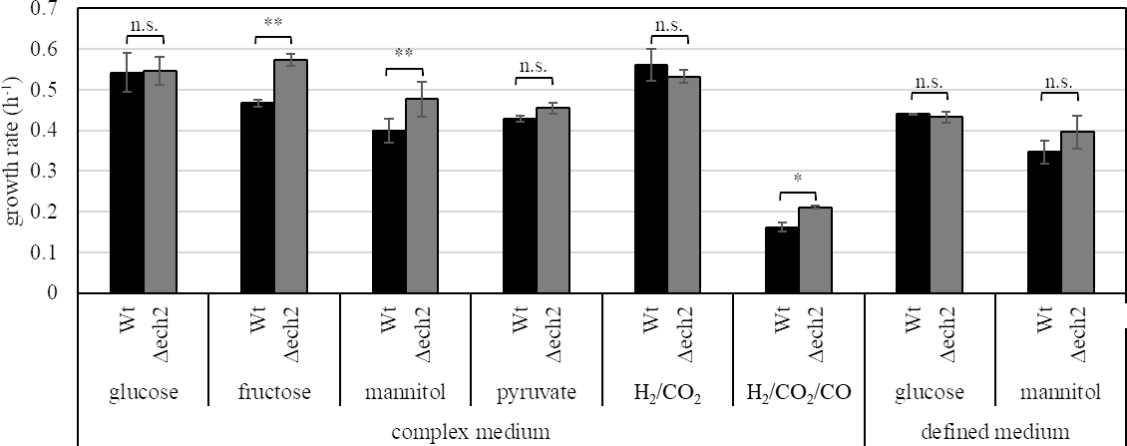
Specific growth rate (h^−1^) of *T. kivui* Δ*ech2* mutant (grey) and the wild type, strain DSM 2030 (black) grown on complex medium containing glucose (*n* = 8), fructose (*n* = 3), mannitol (*n* = 7) (25 mM each) pyruvate (50 mM; *n* = 3), H_2_/CO_2_ (3 atm; 66/33 vol/vol; *n* = 4) or H_2_/CO_2_/CO (3 atm; 44/22/33; vol/vol/vol; *n* = 3) or defined medium containing glucose or mannitol (25 mM each; *n* = 3). The *P*(T <= t) (n.s. = *P* > .05, * =*P* ≤ .05 or ** =*P* ≤ .01) was calculated by a *t*-test (one-tailed and two-sample unequal variance).

**Fig 7:**
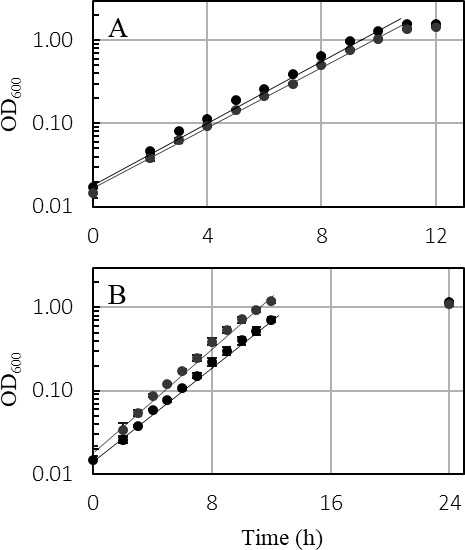
Representative growth curve of the *T. kivui* Δ*ech2* mutant (gray) and the wild type, strain DSM 2030 (black) with (**A**) 25 mM glucose and (**B**) 25 mM mannitol. All experiments were performed on defined medium at 65°C and 160 rpm (*n* = 3).

Growth experiments using different sugars as substrates were performed with the ∆*ech2* mutant and wild type. Interestingly, only slight differences in growth of the ∆*ech2* mutant, compared to the wild type, were observed on glucose, fructose, and the sugar alcohol mannitol. The doubling times of wild type and the ∆*ech2* mutant during growth on complex medium with glucose (1.29 h ± 0.12 *vs*. 1.27 h ± 0.08) (Fig. 4A), fructose (1.49 h ± 0.03 vs. 1.21 h ± 0.03), and mannitol (1.75 h ± 0.14 vs. 1.47 h ± 0.13) (Fig. 5) were similar, with the ∆*ech2* mutant being significantly faster on the latter two substrates (Fig. 6). Consistent with this result, previous studies demonstrated doubling times of 1.24–2.4 h on glucose (24, 25), 1.3 h on fructose (20) and 2 h on mannitol (22) for wild type cells grown on complex medium. Growing cells of wild type and ∆*ech2* mutant metabolized glucose in a homoacetogenic manner, with 2.43 ± 0.23 mol and 2.43 ± 0.28 mol acetate produced from one mol glucose, respectively (Fig. 4B). This corresponds to the previously reported conversion of one mol glucose to 2.5 ± 0.24 mol (26) and 2.6 ± 0.1 mol acetate by *T. kivui* (27). Other acetogens, such as *Moorella thermoacetica* (28) and *Acetobacterium woodii* (29) produce between 2.5 and 2.7 mol acetate per glucose and fructose. In addition to growth experiments on complex medium, growth was also investigated on a defined medium containing uracil. Wild type and ∆*ech2* mutant grown in defined medium with uracil had comparable doubling times on glucose as well (1.57 h ± 0.01, wt; 1.60 h ± 0.05, ∆*ech2*; Fig. 7A) and mannitol (2.01 h ± 0.17, wt; 1.77 h ± 0.20, ∆*ech2*; Fig. 7B). This shows that growth of the ∆*ech2* mutant did not depend on additional substrates or growth factors supplied by the yeast extract in the complex medium. The acetogens *Clostridium ljungdahlii* and *A. woodii* both have an Rnf complex instead of an Ech complex, and deletion of the *rnf* gene cluster had different phenotypes for both species. The ∆*rnf* mutant of *C. ljungdahlii* showed growth inhibition and reduced ATP synthesis on fructose (30). While the ∆*rnf* mutant of *A. woodii* displayed no inhibition of growth on fructose, a change in substrate to acetate ratio was observed. Instead of producing 2.55 mol acetate from one fructose in the wild type, only 2.06 mol acetate was produced, indicating an inhibition of electron flow toward the WLP (31). The ∆*ech2* mutant of *T. kivui*, presented here, did not show any growth inhibition or change in substrate to acetate ratio, as compared to the wild type. Therefore, we conclude that *T. kivui* does not rely on Ech2 during growth on sugars, although the metabolic model suggests that either Ech1 or Ech2 or both are essential for H_2_ oxidation in redox metabolism on sugar substrates (3), as described above.

**Fig 8:**
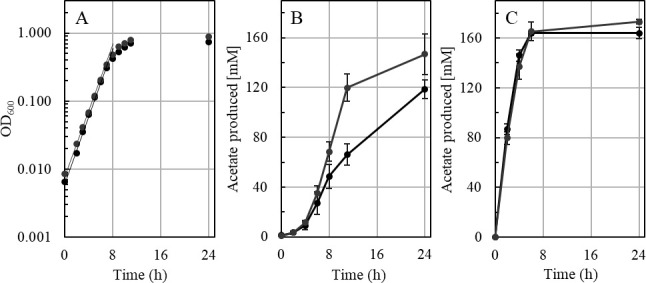
(**A**) Representative growth curve of the *T. kivui* strain Δ*ech2* mutant (gray) and the wild-type strain DSM 2030 (black) in the presence of 3 atm H_2_/CO_2_ (66/33 vol/vol). H_2_/CO_2_ was refilled every hour to a pressure of 3 atm. (**B**) Acetate concentration of wild type (black) and Δ*ech2* mutant (gray) in media of growing cells or (**C**) resting cells. Resting cells were incubated for 24 h in a minimal medium and 3 atm H_2_/CO_2_ (66/33 vol/vol). All experiments were performed on a complex medium at 65°C and 160 rpm. Acetate was determined by gas chromatography (*n* = 4).

For consumption of H_2_ + CO_2_, one or both Ech complexes are assumed to be necessary (Fig. 3A), not only for redox homeostasis but also as the only site for chemiosmotic energy conservation coupled to oxidation of excess Fd_red_ (3, 14, 19). Much to our surprise, the ∆*ech2* mutant grew similar to the wild type on H_2_ + CO_2_ (66/33, vol/vol, 3 atm), with doubling times of 1.24 h ± 0.09 (wild type) vs. 1.30 h ± 0.04 (∆*ech2* mutant) in complex media (Fig. 8A). This growth was faster than the previously reported doubling times of 1.75–2.5 h (26). Interestingly, during growth on H_2_ + CO_2_, acetate is built up at different rates, with the ∆*ech2* mutant accumulating acetate almost twice as fast as the wild type in the exponential phase (10.4 mM h^−1^ ± 1.0, wt; *vs*.17.3 mM h^−1^ ± 2.7, ∆*ech2;* Fig. 8B). Resting cells of the wild type and the ∆*ech2* mutant, however, produced acetate from H_2_ + CO_2_ at a similar rate (Fig. 8C), indicating a higher turnover rate of H_2_ + CO_2_ only during growth. This observation suggests that to compensate for the same growth rate or biomass buildup, the ∆*ech2* mutant consumes more H_2_ than the wild type based on the production of acetate during growth. Higher substrate to product conversion rates and lower yields (Y_substrate_) have been observed for *Escherichia coli* and *Pseudomonas taiwanensis* if respiratory chain complexes such as the cytochrome b oxidases or NADH dehydrogenases are uncoupled (32, 33).

However, this is not easy to explain mechanistically with the current bioenergetic model of *T. kivui* (3), which requires one of the Ech complexes to oxidize Fd_red_ and provide H_2_ to the cell (14). If MetFV is Fd-dependent, coupled to Ech1 and building up a H^+^-gradient, the non-complexed Ech (putatively Ech2) oxidizes H_2_ to provide Fd_red_ to the WLP, at the expense of part of the H^+^-gradient (3) (Fig. 3A). Since we demonstrated that Ech2 is not essential for growth on H_2_ + CO_2_, one option is, that Ech1 is responsible for both Fd_ox_ reduction and energy conservation, however, an Fd-dependent activity of Ech1 has not yet been demonstrated. The second option is depicted in Fig. 3A where H_2_ may be oxidized by HDCR to provide Fd_red_ in addition to formate (34). This, however, is an endergonic reaction. Since HDCR has been found membrane-associated (24), the reaction may be driven by a membrane gradient, although a necessary membrane-spanning motif has not been demonstrated as part of the complex. If HDCR runs in reverse and produces Fd_red_, this would benefit energy conservation which would be inconsistent with the ∆*ech2* mutant producing less biomass per acetate.

### Growth on Fd-dependent substrates is impaired

In addition to sugar and H_2_ + CO_2_, *T. kivui* can grow on pyruvate (26), and it has been adapted to grow on 100% CO (35). In contrast to all other substrates tested, pyruvate and carbon monoxide (CO) are substrates whose oxidation solely provides Fd_red_. While pyruvate is oxidized by pyruvate:Fd oxidoreductase (POR) in *T. kivui* (7) there are two CO dehydrogenases in *T. kivui* that catalyze a Fd-dependent CO oxidation, CODH/ACS, and the monofunctional CooS (36). The latter has been demonstrated to be responsible for CO oxidation during growth on the gas (36), whereas the CODH/ACS as the key enzyme in the WLP, mainly reduces CO_2_ to CO (4). Fd_red_ from CO or pyruvate oxidation would have to be re-oxidized by either of the Ech complexes depending on the assumed pathway model (7, 14). Under the assumption that Ech1 cannot oxidize Fd_red_, deletion of Ech2 should lead to a redox imbalance and should thus inhibit growth on CO.

**Fig 9:**
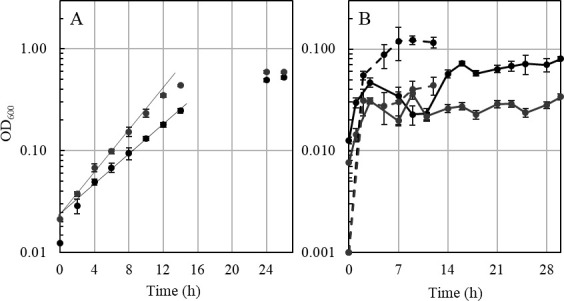
(**A**) Representative growth curve of ∆*ech2* mutant on H_2_/CO_2_/CO. Growth of the wild type (black) and ∆*ech2* mutant (gray) in the presence of 3 atm H_2_/CO_2_/CO. All experiments were performed on complex medium at 65°C and 160 rpm. Gas mix H_2_/CO_2_/CO (44/22/33; vol/vol/vol; [2 atm H_2_/CO_2_ (66/33; vol/vol) plus 1 atm pure CO]). (**B**) Increase of OD_600_ of Δ*ech2* mutant and the wild-type strain, DSM 2030 in the presence of CO/N_2_ (3 atm; 14/86 vol/vol). The Δ*ech2* mutant and the wild type were inoculated with an OD_600_ of around 0.01 (passage 1; solid line), while passage 2 (dashed line) was inoculated with an OD_600_ of 0.001 after 23 days from passage 1. All experiments were performed on complex medium at 65°C and 160 rpm (*n* = 3).

In accordance with this logic, we recently demonstrated that resting cells of the ∆*ech2* mutant are unable to produce acetate from CO (37). To study the effect of the *ech2* deletion on growth with CO, we tried to adapt the ∆*ech2* mutant to CO. For the adaption of *T. kivui* for growth on CO as the only substrate, several weeks are required (35). Therefore, wild type and ∆*ech2* mutant were first cultured on H_2_/CO_2_ in addition to CO (3 atm; 44/22/33; vol/vol/vol) (Fig. 9A). Subsequently, the cells were transferred to CO/N_2_ atmosphere (3 atm; 14/86; vol/vol) (Fig. 9B), where they were cultured for several days. The wild type grew to an OD_600_ of 0.057 after 14 days, whereas the ∆*ech2* mutant remained at 0.023. To test the adaption and viability of the cells, wild type and ∆*ech2* mutant were passaged after 23 days. 7 days after the transfer, the wild type had grown to an OD_600_ of 0.12 while the ∆*ech2* mutant stopped growing at an OD_600_ of around 0.04 (Fig. 7B). This low increase in OD_600_ of the ∆*ech2* mutant is most likely due to the yeast extract in the medium, which can be utilized by *T. kivui* (25). From these findings, we concluded that *T. kivui* depends on Ech2 for catabolic conversion and growth on CO.

**Fig 10:**
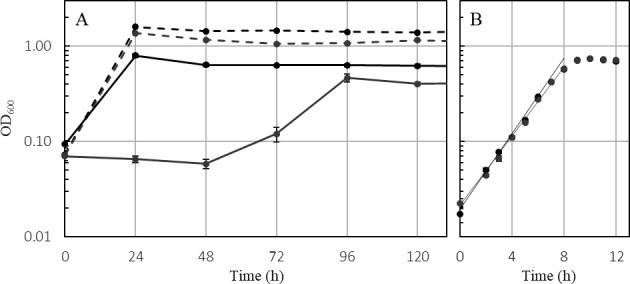
(**A**) First transfer of the Δ*ech2* mutant and wild type from glucose to pyruvate. Growth of the *T. kivui* strain Δ*ech2* mutant (gray) and the wild-type strain DSM 2030 (black) in the presence of 50 mM pyruvate (continuous line) or 25 mM glucose (dashed line). The experiment was performed in Hungate tubes with 5 mL complex medium at 65°C. (**B**) Growth of the *T. kivui* Δ*ech2* mutant (gray) and the wild type, strain DSM 2030, (black) in the presence of 50 mM pyruvate (fourth transfer on pyruvate). All experiments were performed on a complex medium at 65°C and 160 rpm (*n* = 3±SD).

**Fig 11:**
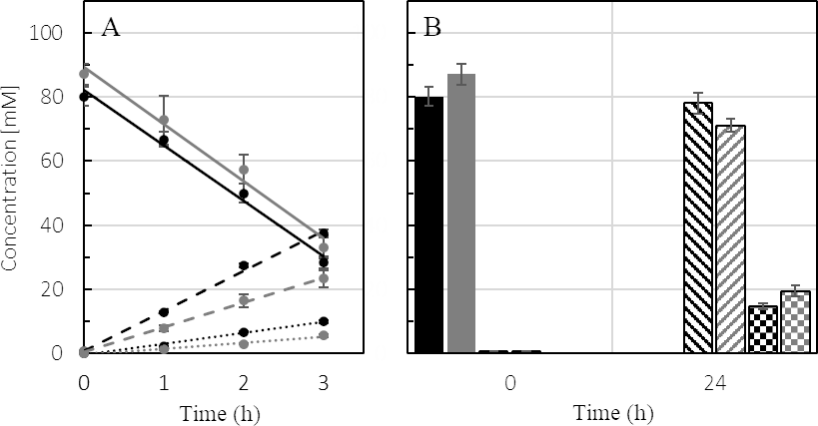
Resting cell experiment of the wild type (black) and *ech2* mutant (gray). Incubated for 24 h in Tris-HCL buffer with ca. 100 mM pyruvate at 65 C. (**A**) Concentration changes in pyruvate (solid line), acetate (dashed line), and formate (dotted line) over 3 h. (**B**) Concentration at the beginning and after 24 h of pyruvate (filled column), acetate (striped column), and formate (tiled column). Acetate and formate concentrations at the beginning of the experiment were below 0.75 mM, while almost all pyruvate was consumed within 24 h.

To test growth on the second Fd-dependent substrate, pyruvate, cultures of *T. kivui* wild type and ∆*ech2* mutant were grown on glucose and subsequently transferred to complex medium with pyruvate as the only substrate. The wild type grew immediately after inoculation, whereas the ∆*ech2* mutant did not grow for 48 to 72 h after inoculation, but ultimately reached an OD_600_ of 0.464 ± 0.045 after 96 h. As a control, we inoculated the ∆*ech2* mutant on a medium with glucose at the same time and with this substrate, the strain grew immediately (Fig. 10A). After four passages on pyruvate, however, the ∆*ech2* mutant showed a similar doubling time as the wild type (wt, 1.61 h ± 0.03; 1.53 h ± 0.05, ∆*ech2* mutant) (Fig. 10B). Interestingly, resting cells of the wild type and the ∆*ech2* mutant that were not previously adapted to pyruvate, were able to utilize pyruvate at a similar rate (−17.23 mM/h, wt vs. −17.72 mM/h, ∆*ech2*) (Fig. 11A), but with different acetate (+12.45 mM/h, wt; vs. +7.68 mM/h, ∆*ech2* mutant) and formate (+3.43 mM/h, wt; +1.84 mM/h ∆*ech2* mutant) production rates. After 24 h, the wild type and ∆*ech2* mutant had consumed 80.16 ± 2.90 mM and 87.11 ± 3.24 mM pyruvate, respectively, and produced 78.09 ± 3.30 mM (wt) and 71.05 ± 2.01 mM (∆*ech2*) acetate, respectively. In addition to acetate, the wild type produced 14.73 ± 0.86 mM formate, while the ∆*ech2* mutant produced 19.50 ± 1.80 mM formate after 24 h (Fig. 11B). Of interest here is that for the production of 1 mM acetate the wild type required 1.02 mM pyruvate, whereas the ∆*ech2* mutant required 1.23 mM of pyruvate, indicating a reduced electron flux toward the WLP. The carbon and electron balance during pyruvate conversion was not closed. This change in acetate yield from pyruvate was likely due to an unidentified product. This was previously observed by Leigh et al. (26), who reported that 0.93 mM pyruvate was necessary to produce 1 mM acetate in the wild type.

**Fig 12:**
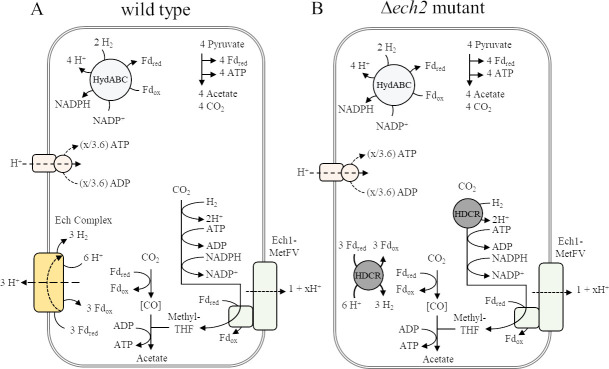
Model of acetogenesis from pyruvate in *Thermoanaerobacter kivui* (**A**) wild type (modified from (3)) and (**B**) *ech2* mutant. (**A**) pyruvate oxidation provides the required reducing equivalents for the reduction of CO_2_ in the WLP. The electron-bifurcating hydrogenase (HydABC) oxidizes H_2_ to provide Fd_red_ and NADPH and the energy-conserving hydrogenase (Ech complex) oxidize Fd_red_ to Fd_ox_ and reduce H^+^ to H_2_. (**B**) Hypothetical replacement of Ech2 redox activities by HDCR. Ech1-MetFV is assumed to translocate 1 + x H^+^ across the membrane. The generated proton motive force is used to synthesize ATP *via* ATP synthase.

These data demonstrate that *T. kivui* depends on Ech2 for the oxidation of Fd_red_, to a different extent for different substrates. Neither Ech1 nor any other enzyme may substitute for Ech2 for CO utilization. Meanwhile, during growth on pyruvate, substrate conversion is slowed down and growth is initially impaired, but—putatively through a mutation in the genome—the ∆*ech2* mutant may be adapted to grow on pyruvate. During growth on H_2_ + CO_2_ or sugars, no growth inhibition, not even initially, was observed. This aggregate of observations implies that there must be another enzyme involved in Fd_red_ oxidation coupled to H_2_ production. Importantly, this activity has not been demonstrated for Ech1 (Volker Müller, pers. communication), and therefore, we propose that the alternative activity of HDCR may substitute for Ech2 (Fig. 12). The HDCR of *T. kivui* is described to oxidize H_2_, or Fd_red_ provided by CODH during CO consumption to fix CO_2_; thus, the HCDR could oxidize Fd_red_ to reduce H^+^ (10, 34). It is unclear, why this affects pyruvate metabolism more than sugar and H_2_ + CO_2_ metabolism since the predicted direction of Ech2 is Fd_ox_ reduction with the latter two substrates (3), which is thermodynamically more difficult.

### Adaptation to CO involves a conserved SNP in HDCR

It is also unclear, why CO metabolism is completely inhibited, while growth on pyruvate is only initially impaired. Putatively, CODH may form an essential complex with Ech2 (23), or HDCR, that is CO sensitive, may carry a mutation toward a lower CO sensitivity, simultaneously affecting its interaction with Fd. To investigate this hypothesis, we performed a SNP analysis on the *T. kivui* strain originally adapted to CO (35), as well as on a strain adapted to CO in our hands. A ∆*pyrE* CO-adapted strain with a large horizontal gene transfer has already been described (38). In our laboratory, the wild type was re-adapted to CO, as described originally in Weghoff (35), and four single colonies from the adapted culture were picked and sent for SNP sequencing. Sequencing results from an additional strain, adapted to low-temperature growth rather than to CO (39; data set available in the Sequence Read Archive (SRA) under the accession number SRR25301688), were included to identify mutations already present in the laboratory’s wild-type strain, or resulting from adaptations to the media/culture conditions used in the laboratory, rather than specifically to the presence of CO. Reads from the original sequencing run (35) were re-analyzed using the same BreSeq analysis pipeline as the other samples to rule out spurious alignment artifacts.

The ∆*pyrE*-CO strain had 313 identified SNPs, but most (265) were in the area of the previously characterized HGT and were removed for this analysis. All other strains had around 60 identified SNPs. After filtering out all SNPs present in the re-analyzed wild type or in the low temperature adapted strain (un-related to CO-adaptation), and after removing all silent and all intergenic mutations more than 100 bp upstream of a gene, a total of 69 unique SNPs remained that could be associated with the CO-adapted phenotype. A subset of the most interesting SNPs is shown in Table 2. Evidently, a high concentration of mutations was present in *acsA* and *cooC*, two genes important for the function of the CODH/ACS complex. Two distinct mutations result in almost complete disruption of the CooC protein, while the two other mutations lead to single amino acid substitutions near the N-terminus. All three mutations present in the *acsA* gene lead to early frame shifts disrupting the resulting protein, but each mutation is only present in a small subset of the population (between 5% and 20% of reads). A previous study in strains of the acetogen *Eubacterium limosum* following adaptation to CO found almost identical disruption mutations in *cooC2*, which the authors believe are possible because *cooC* is non-essential when nickel is plentiful, as is the case in most synthetic laboratory media (40). However, the complete disruption of *acsA* observed in our data is surprising since the CODH/ACS complex is essential for acetogenesis. Adapted strains of *E. limosum* also exhibited mutations in *acsA* (40), and both *acsA* and *acsB* (41), but in all cases these were single amino acid substitutions. That three *acsA* disruption mutations occurred independently in our adapted strains suggests they do confer some advantage during growth on CO but the low prevalence in the population (low percentage of reads) could be indicative of a balance between whatever benefit the mutations provide and the detrimental effect of disrupting such an important gene.

**TABLE 2:** Selected SNPs detected in *T. kivui* strains adapted to CO, and the percent of reads in each strain that contains a given SNP

Position (Bp)	Mutation	WT CO-1	WT CO-2	WT CO-3	WT CO-4	∆*pyrE* CO	Amino acid change	Gene [plus (→) or minus (←) strand]	Annotation
129,729	T→C					100%	L124P (CTA→CCA)	*ech1C* →	Ech-type complex subunit Ech1C
1,883,965	Δ1 bp					78%	Coding (1126/1716 nt)	*hydA1* ←	Electron-bifurcating H_2_ase subunit HydA1
1,899,612	G→T					100%	H109N (CAT→AAT)	*ech2E* ←	Ech-type complex subunit Ech2E
1,900,320	G→C					100%	Intergenic (−16/+334)	*ech2D* ← / ← Hyp	Ech-type complex subunit Ech2D/hypothetical
1,922,082	T→C					33%	E67G (GAG→GGG)	*fdhD* ←	HDCR subunit FdhD
1,924,564:1	+A		100%	100%	100%		Coding (505/555 nt)	*hycB3* ←	HDCR subunit HycB3
1,924,576	+TTTA					100%	Coding (493/555 nt)	*hycB3* ←	HDCR subunit HycB3
1,924,783	C→T	100%					G96R (GGG→AGG)	*hycB3* ←	HDCR subunit HycB3
1,935,292	Δ1 bp				100%		Coding (368/777 nt)	*cooC2* ←	CODH accessory protein CooC
1,935,633	Δ1 bp		42%				Coding (27/777 nt)	*cooC2* ←	CODH accessory protein CooC
1,935,640	C→T	11%	7%				G7E (GGA→GAA) ‡	*cooC2* ←	CODH accessory protein CooC
1,935,641	C→T	23%					G7R (GGA→AGA) ‡	*cooC2* ←	CODH accessory protein CooC
1,936,088	Δ1 bp			6%			Coding (1516/1857 nt)	*acsA* ←	CODH catalytic subunit acsA
1,937,286	Δ1 bp	16%					Coding (318/1857 nt)	*acsA* ←	CODH catalytic subunit acsA
1,937,567	T→A		21%				K13* (AAA→TAA)	*acsA* ←	CODH catalytic subunit acsA
2,134,417	Δ8 bp					100%	Coding (1200/1224 nt)	*nfnB* →	Transhydrogenase subunit B

Given the known toxicity of CO to the metal centers of hydrogenases, it was unsurprising to see mutations in genes for the electron-bifurcating hydrogenase (*hydA1*), subunits of the energy-converting hydrogenases Ech1 (*ech1C*) and Ech2 (*ech2E* and promoter of *ech2D*), as well as subunits of the hydrogen-dependent carbon dioxide reductase (*fdhD* and *hycB3*), and the transhydrogenase (*nfnB*) (Table 2). As with the *cooC* gene, there are several frame shifts, but most disrupt only the C-terminus of the relevant gene. For example, two independent mutations remove roughly the last 10% of amino acids from HycB3, and one mutation disrupts only 2% (the last eight amino acids) of NfnB. The largest disruption among the hydrogenases is in HydA1, where two-thirds of the protein remains intact.

The mutations leading to disruption of the C-terminus of HycB3 are of particular interest. The mutations identified here introduce frame shifts at codons 164 and 168 of HycB3, while previous research found that terminating the protein at codon 160 disrupts the oligomeric structure of the HDCR complex, and results in a single hetero-hexametric subunit that is still functional but has reduced activity (to 33% of the wild-type’s activity for formate production from H_2_ + CO_2_, and 18% for H_2_ production from formate) (24, 34). HycB3 contains four 4Fe-4S clusters for transferring electrons within the HDCR complex, but the C-terminal truncations described here come after the iron-sulfur coordinating cysteine residues.

As mentioned previously, we hypothesize that a side reaction of the HDCR allows for partial compensation for the loss of Fd_red_ re-oxidation normally carried out by Ech2 during growth on pyruvate. It seems likely that the disruption of the HDCR oligomeric complex caused by the HycB3 mutation in the CO-adapted strains also disrupts this side reaction. If this mutation is essential for CO-adaptation (and it is worth noting that all five sequenced CO-adapted strains had a mutation in *hycB3*), then growth on CO and deletion of Ech2 are mutually exclusive: the Ech2 mutant requires the oligomeric HDCR side reaction to re-oxidize Fd, while the CO-adapted strains require its monomeric form (possibly because of a structural shift that protects from CO-toxicity). Since the *hycB3* disruption is not necessary for growth on pyruvate, the cells are eventually able to grow on pyruvate, although after a substantial lag phase.

## MATERIAL AND METHODS

### Growth conditions

*Thermoanaerobacter kivui* strains were routinely grown anaerobically at 65°C in a modified DSMZ171 medium. Defined medium contained 50 mM Na_2_HPO_4_, 50 mM NaH_2_PO_4_, 1.3 mM K_2_HPO_4_, 1.6 mM KH_2_PO_4_, 3.8 mM NaCl, 2.9 mM NH_4_Cl, 0.8 mM (NH_4_)_2_SO_4_, 0.4 mM MgSO_4_, 3.6 µM FeSO_4_, 5.6 µM CaCl_2_, 1% trace element solution DSMZ141, 1% mL vitamin solution DSMZ141. The complex medium additionally contained 2 g L^−1^ yeast extract (AppliChem GmbH, Darmstadt, Germany). Preparation of the medium was performed as previously described (20). Media was purged with Protadur C20 [80% / 20% (vol/vol) N_2_/CO_2;_ Westfalen AG, Münster] and autoclaved. If not described otherwise, cells were grown with 25 mM glucose, 25 mM mannitol or 25 mM fructose or 50 mM or 100 mM pyruvate as substrate, each added from sterile, anoxic stock solutions. Autotrophic cultures were grown on H_2_/CO_2_ [66%/33%; (vol/vol); 2 × 10^5^ Pa, Westfalen AG, Münster] or carbon monoxide (2 × 10^5^ Pa, Westfalen AG, Münster). If not described otherwise, all growth experiments were carried out on complex medium under strict anoxic conditions using 100 mL or 200 mL serum bottles filled with 40 mL medium, and sealed with butyl rubber stoppers. For all growth experiments, cultures were inoculated from pre-cultures grown on the same substrate. Growth was monitored by measuring the absorption at 600 nm (OD_600_).

For transformation, *T. kivui* was cultured as described previously (20). Defined medium agar with 1.5% Bacto agar BD (Difco, BD Life Sciences, Heidelberg, Germany) was prepared, autoclaved, and cooled down below 65°C to add substrates. If required, uracil or 5-fluoroorotic acid (5-FOA) was supplemented to minimal medium agar, and the agar is then poured into 20 mL petri dishes containing a cell suspension. The petri dishes holding liquid agar were transferred into an anoxic box (Coy Laboratory Products, Grass Lake, MI) with a former gas H_2_/N_2_ [5%/95%; (vol/vol)] atmosphere to solidify. The petri dishes were put into an incubator jar containing a palladium catalyst and calcium chloride as water absorbent. The jar was sealed inside the anoxic box, and the gas atmosphere was adjusted to an overpressure of 5 × 10^4^ Pa with Protadur C20 [80%/20% (vol/vol) N_2_/CO_2_]. The jar was transferred for incubation at 65°C. After the incubation period, the jar was taken out and allowed to cool down to room temperature before opening.

### Genetic manipulation of *Thermoanaerobacter kivui*

For generation of the *ech2* mutant strain, an integrating plasmid pEch2Tk02 was constructed according to Basen et al. (20). The plasmid is derived from pMBTKv0022, which enables markerless deletions of genes *via* two independent homologous recombination events. 0.5 kbp upstream (UFR) and downstream (DFR) flanking regions of the *ech2* gene cluster (TKV_c19680–TKV_c19750) were amplified, using the primer pairs 2E02a/2E02b and 2E02c/2E02d (Fig. 1). Both fragments were fused *via* PCR using the primers 2E02a/2E02d. The resulting UFR/DFR fragment and pMBTKv0022 were digested with BamHI and XbaI. Plasmid and fragment were ligated *via* T4 ligase, and the resulting plasmid pE2TK02 was transformed into the ∆*pyrE* strainTKV_MB002 (20, 25). Two selection rounds were performed to generate ∆*ech2* according to 20 (20). First, the transformants were plated on a minimal medium agar (1.5%) without uracil to enforce the integration of the plasmid encoding *pyrE* and the UFR and DFR. Grown colonies were transferred to minimal medium. To enforce the loss of the plasmid encoding *pyrE* (including *ech2*), 5 mM 5-FOA and 40 mM uracil were supplemented to minimal medium agar. Grown uracil-auxotrophic colonies were transferred into a minimal medium containing 50 mM uracil and screened by PCR for the loss of *ech2* using primers O2a/O2d afterward. The loss of the *ech2* operon was verified by qPCR.

### Resting cell experiment

Resting cells of *T. kivui* were prepared as described previously (37) under strict anoxic conditions in an anoxic glove box (Coy Laboratory Products, Grass Lake, MI) containing a reducing gas atmosphere [H_2_/N_2_; 5%/95%; (vol/vol)] atmosphere. Cells were cultivated in 250–500 mL complex medium in 1 L bottles (SCHOTT AG, Mainz, Germany), and harvested in the late exponential growth phase by centrifugation at 4,000 rpm, 4°C for 15 min. The cells were then first washed in imidazole buffer (50 mM imidazole, 20 mM MgSO4, 20 mM KCl, 4 µM resazurin, 2 mM DTE, pH 7.0), subsequently resuspended in imidazole buffer to a final OD**_600_** of 2 and finally transferred to 50 mL serum bottles, which were then sealed with butyl rubber stoppers. The gas H_2_/N_2_ atmosphere [5%/95%; (vol/vol)] in the serum bottles was replaced by flushing with N_2_ gas to remove the H_2_. Finally, the headspace was changed to CO/N_2_ [2 × 10^5^ Pa, 20/80 (vol/vol)] in the experiment toward CO conversion, and to CO_2_/N_2_ [1.1 × 10^5^ Pa, 20/80 (vol/vol)] in the experiment toward pyruvate conversion.

### Metabolite analysis

The concentrations of glucose and pyruvate, formate, and acetate were determined by high-performance liquid chromatography. For the sample preparation, cells were centrifugated at 13,000 rpm for 10 min at 4°C and 250 µL of supernatant was filled into a 1.5 mL polypropylene tube and 5 µL H_2_SO_4_ (50%) was added to the supernatant. The sample was mixed and centrifuged at 13,000 rpm for 10 min at 4°C. Afterward, 200 µL of the sample was transferred into an HPLC vial. Separation of sugars, alcohols, and organic acids was performed using a 300 × 8 mm column packed with organic acid resin (CS—Chromatography Service GmbH, Langerwehe, Germany) on a Shimadzu (Kyoto, Japan) LC20 system, equipped with a SIL-20AC autosampler (10 µL sample injection), a LC-20AD pump a CTO-20AC column oven (30°C), RID-10A refractive index detector and a UV-Vis detector, at a flow rate of 0.6 mL/min (5 mM H_2_SO_4_ as eluent).

Acetate concentrations were determined by gas chromatography. For the sample preparation, cells were centrifuged at 13,000 rpm for 10 min at 4°C and 450 µL of supernatant was filled into a 1.5 mL polypropylene tube and 50 µL 2 M phosphor acid was added to the supernatant. The sample was mixed and centrifuged at 13,000 rpm for 10 min at 4°C. Next, 450 µL of the supernatant was mixed with 50 µL of 2 M phosphoric acid and 550 µL of H20_dd_ and transferred to a GC vial. Gas chromatography was performed using an 8860 GC System (Agilent, Santa Clara, United States), equipped with a 6 Ft 1/8 2 mm HayeSep P 60/80 UM column and a flame ionization detector (FID). The FID operates at 250°C, with an airflow (synthetic air) of 300 mL/min, an H2 flow of 30 mL/min, and a make-up flow (N_2_) of 25 mL/min. As carrier gas served pure N_2_ (Westfalen AG, Münster, Germany) at a flow rate of 10 mL/min. 0.5 µL of sample was injected at 195°C using a PPZ inlet with a flow rate of 10 mL/min. A temperature gradient was applied by starting from 120°C for 3 min followed by an increase of 5 °C/min to 145°C for 3 min, followed by an increase of 10 °C/min to 170°C for 0.5 min. In the last step, the temperature was increased by 30 °C/min to 200°C. Data analysis was performed with the OpenLAB CDS EZChrom software (Agilent, Santa Clara, United States).

### RNA extraction and qPCR

RNA extraction was performed as described in the protocol of the RNeasy Protect Kit and RNase-Free DNase Set (QIAGEN GmbH, Hilden, Germany). A cDNA Synthesis Kit (Biozym Scientific GmbH, Hessisch Oldendorf, Germany) was used to generate cDNA and the following qPCR was run with Blue S'Green qPCR Mix Separate ROX (Biozym Scientific GmbH, Hessisch Oldendorf, Germany) and analyzed with qTOWER³ (Analytik Jena GmbH, Jena, Germany). Primers (Table 3) used in this study were ordered from Merck KGaA (Darmstadt, Germany). The housekeeping gene for qPCR was *gyr*AB.

**TABLE 3:** Primers used in this study

Primer	Sequence 5′-3′	Application
2E02a	CCCGGGGATCCTTGAAGAGTATGCCAACATGTGTTATT	Cloning construct pEch2TK02
2E02b	AAATCATATTGGTGGGGGTGGGTAAGAAC	Cloning construct pEch2TK02
2E02c	CACCCCCACCAATATGATTTGTCCCAAACCCTATATCTACA	Cloning construct pEch2TK02
2E02d	CAGGTCGACTCTAGAATTTTTCAATGGCAAGAAAAAGATTAACGT	Cloning construct pEch2TK02
O2a	CAATGCATTTTGCTAAGTTTAATGTATATTTTGA	
O2d	TGCAGATGTTTATTTTTCAATGGCAA	
gyrB_for_qRT	CCAGT TGTGC TTCCT TCTCG ATTTC C	qRT-PCR
gyrB_rev_qRT	GCGAC AATGC CATCT ATGAC TTCTC C	qRT-PCR
BZq25 (Ech2)	GAGGTTCAATTGAAGCCTGAGATG	qRT-PCR
BZq26 (Ech2)	CAGCAGAATGGGCAGAAAGAG	qRT-PCR
Ech1A for qRT T.k.	CCTCCTTTGCCGGTGTAATGAGTAAGG	qRT-PCR
Ech1A rev qRT T.k.	AAGCATGGTAAACGCACCCAAC	qRT-PCR

### Single nucleotide polymorphism analysis

The genomes of adapted strains were analyzed for single nucleotide polymorphisms (SNPs) as recently described (38).

### Statistical analysis

Data sets were analyzed by *t*-tests (one-tailed and two-sample unequal variance) using the software Microsoft Excel (Microsoft Corporation, Redmond, Washington, USA).

## Data Availability

The datasets generated for this study have been deposited in the Sequence Read Archive (SRA) under the accession number (SRR25949164-SRR25949167).

## References

[1] Drake HL, Gössner AS, Daniel SL. 2008. Old acetogens, new light. Ann N Y Acad Sci 1125:100–128. doi:10.1196/annals.1419.016 18378590

[2] Müller V. 2003. Energy conservation in acetogenic bacteria. Appl Environ Microbiol 69:6345–6353. doi:10.1128/AEM.69.11.6345-6353.2003 14602585 PMC262307

[3] Katsyv A, Jain S, Basen M, Müller V. 2021. Electron carriers involved in autotrophic and heterotrophic acetogenesis in the thermophilic bacterium Thermoanaerobacter kivui. Extremophiles 25:513–526. doi:10.1007/s00792-021-01247-8 34647163 PMC8578170

[4] Ragsdale SW, Pierce E. 2008. Acetogenesis and the Wood-Ljungdahl pathway of CO_2_ fixation. Biochim Biophys Acta 1784:1873–1898. doi:10.1016/j.bbapap.2008.08.012 18801467 PMC2646786

[5] Schuchmann K, Müller V. 2014. Autotrophy at the thermodynamic limit of life: a model for energy conservation in acetogenic bacteria. Nat Rev Microbiol 12:809–821. doi:10.1038/nrmicro3365 25383604

[6] Basen M, Müller V. 2017. "Hot" acetogenesis. Extremophiles 21:15–26. doi:10.1007/s00792-016-0873-3 27623994

[7] Katsyv A, Schoelmerich MC, Basen M, Müller V. 2021. The pyruvate:ferredoxin oxidoreductase of the thermophilic acetogen, Thermoanaerobacter kivui. FEBS Open Bio 11:1332–1342. doi:10.1002/2211-5463.13136 PMC8091585 33660937

[8] Mock J, Wang S, Huang H, Kahnt J, Thauer RK. 2014. Evidence for a hexaheteromeric methylenetetrahydrofolate reductase in Moorella thermoacetica. J Bacteriol 196:3303–3314. doi:10.1128/JB.01839-14 25002540 PMC4135698

[9] Mock J, Zheng Y, Mueller AP, Ly S, Tran L, Segovia S, Nagaraju S, Köpke M, Dürre P, Thauer RK. 2015. Energy conservation associated with ethanol formation from H_2_ and CO_2_ in Clostridium autoethanogenum involving electron bifurcation. J Bacteriol 197:2965–2980. doi:10.1128/JB.00399-15 26148714 PMC4542177

[10] Schuchmann K, Müller V. 2012. A bacterial electron-bifurcating hydrogenase. J Biol Chem 287:31165–31171. doi:10.1074/jbc.M112.395038 22810230 PMC3438948

[11] Biegel E, Müller V. 2010. Bacterial Na^+^-translocating ferredoxin:NAD^+^ oxidoreductase. Proc Natl Acad Sci U S A 107:18138–18142. doi:10.1073/pnas.1010318107 20921383 PMC2964206

[12] Kuhns M, Trifunović D, Huber H, Müller V. 2020. The Rnf complex is a Na^+^ coupled respiratory enzyme in a fermenting bacterium, Thermotoga maritima. Commun Biol 3:431. doi:10.1038/s42003-020-01158-y 32770029 PMC7414866

[13] Schuchmann K, Müller V. 2016. Energetics and application of heterotrophy in acetogenic bacteria. Appl Environ Microbiol 82:4056–4069. doi:10.1128/AEM.00882-16 27208103 PMC4959221

[14] Hess V, Poehlein A, Weghoff MC, Daniel R, Müller V. 2014. A genome-guided analysis of energy conservation in the thermophilic, cytochrome-free acetogenic bacterium Thermoanaerobacter kivui. BMC Genomics 15:1139. doi:10.1186/1471-2164-15-1139 25523312 PMC4320612

[15] Meuer J, Kuettner HC, Zhang JK, Hedderich R, Metcalf WW. 2002. Genetic analysis of the archaeon Methanosarcina barkeri Fusaro reveals a central role for Ech hydrogenase and ferredoxin in methanogenesis and carbon fixation. Proc Natl Acad Sci U S A 99:5632–5637. doi:10.1073/pnas.072615499 11929975 PMC122822

[16] Sapra R, Bagramyan K, Adams MWW. 2003. A simple energy-conserving system: proton reduction coupled to proton translocation. Proc Natl Acad Sci U S A 100:7545–7550. doi:10.1073/pnas.1331436100 12792025 PMC164623

[17] Hedderich R, Hamann N, Bennati M. 2005. Heterodisulfide reductase from methanogenic archaea: a new catalytic role for an iron-sulfur cluster. Biol Chem 386:961–970. doi:10.1515/BC.2005.112 16218868

[18] Welte C, Krätzer C, Deppenmeier U. 2010. Involvement of Ech hydrogenase in energy conservation of Methanosarcina mazei. FEBS J 277:3396–3403. doi:10.1111/j.1742-4658.2010.07744.x 20629748

[19] Schoelmerich MC, Müller V. 2019. Energy conservation by a hydrogenase-dependent chemiosmotic mechanism in an ancient metabolic pathway. Proc Natl Acad Sci U S A 116:6329–6334. doi:10.1073/pnas.1818580116 30850546 PMC6442639

[20] Basen M, Geiger I, Henke L, Müller V, Elliot MA. 2018. A genetic system for the thermophilic acetogenic bacterium Thermoanaerobacter kivui. Appl Environ Microbiol 84:e02210-17. doi:10.1128/AEM.02210-17 29150512 PMC5772241

[21] Katsyv A, Müller V. 2022. A purified energy-converting hydrogenase from Thermoanaerobacter kivui demonstrates coupled H^+^-translocation and reduction in vitro. J Biol Chem 298:102216. doi:10.1016/j.jbc.2022.102216 35779632 PMC9356269

[22] Moon J, Henke L, Merz N, Basen M. 2019. A thermostable mannitol-1-phosphate dehydrogenase is required in mannitol metabolism of the thermophilic acetogenic bacterium Thermoanaerobacter kivui. Environ Microbiol 21:3728–3736. doi:10.1111/1462-2920.14720 31219674

[23] Schuchmann K, Müller V. 2013. Direct and reversible hydrogenation of CO_2_ to formate by a bacterial carbon dioxide reductase. Science 342:1382–1385. doi:10.1126/science.1244758 24337298

[24] Dietrich HM, Righetto RD, Kumar A, Wietrzynski W, Trischler R, Schuller SK, Wagner J, Schwarz FM, Engel BD, Müller V, Schuller JM. 2022. Membrane-anchored HDCR nanowires drive hydrogen-powered CO_2_ fixation. Nature 607:823–830. doi:10.1038/s41586-022-04971-z 35859174

[25] Jain S, Dietrich HM, Müller V, Basen M. 2020. Formate is required for growth of the thermophilic acetogenic bacterium Thermoanaerobacter kivui lacking hydrogen-dependent carbon dioxide reductase (HDCR). Front Microbiol 11:59. doi:10.3389/fmicb.2020.00059 32082286 PMC7005907

[26] Leigh JA, Mayer F, Wolfe RS. 1981. Acetogenium Kivui, a new thermophilic hydrogen-oxidizing acetogenic bacterium. Arch Microbiol 129:275–280. doi:10.1007/BF00414697

[27] Moon J, Jain S, Müller V, Basen M. 2020. Homoacetogenic conversion of mannitol by the thermophilic acetogenic bacterium Thermoanaerobacter kivui requires external CO_2_. Front Microbiol 11:571736. doi:10.3389/fmicb.2020.571736 33042077 PMC7522397

[28] Fontaine FE, Peterson WH, McCoy E, Johnson MJ, Ritter GJ. 1942. A new type of glucose fermentation by Clostridium thermoaceticum. J Bacteriol 43:701–715. doi:10.1128/jb.43.6.701-715.1942 16560531 PMC373636

[29] Heise R, Müller V, Gottschalk G. 1989. Sodium dependence of acetate formation by the acetogenic bacterium Acetobacterium woodii. J Bacteriol 171:5473–5478. doi:10.1128/jb.171.10.5473-5478.1989 2507527 PMC210386

[30] Tremblay P-L, Zhang T, Dar SA, Leang C, Lovley DR. 2012. The Rnf complex of Clostridium ljungdahlii is a proton-translocating ferredoxin:NAD^+^ oxidoreductase essential for autotrophic. mBio 4:e00406-12. doi:10.1128/mBio.00406-12 23269825 PMC3531802

[31] Westphal L, Wiechmann A, Baker J, Minton NP, Müller V. 2018. The Rnf complex is an energy-coupled transhydrogenase essential to reversibly link cellular NADH and ferredoxin pools in the acetogen Acetobacterium woodii. J Bacteriol 200:e00357-18. doi:10.1128/JB.00357-18 30126940 PMC6182241

[32] Bekker M, de Vries S, Ter Beek A, Hellingwerf KJ, de Mattos MJT. 2009. Respiration of Escherichia coli can be fully uncoupled via the nonelectrogenic terminal cytochrome bd-II oxidase. J Bacteriol 191:5510–5517. doi:10.1128/JB.00562-09 19542282 PMC2725625

[33] Nies SC, Dinger R, Chen Y, Wordofa GG, Kristensen M, Schneider K, Büchs J, Petzold CJ, Keasling JD, Blank LM, Ebert BE. 2020. Systems analysis of NADH dehydrogenase mutants reveals flexibility and limits of Pseudomonas taiwanensis VLB120's metabolism. Appl Environ Microbiol 86:e03038-19. doi:10.1128/AEM.03038-19 32245760 PMC7237778

[34] Dietrich HM, Müller V. 2023. Ferredoxin as physiological electron donor for carbon dioxide fixation to formate in a bacterial carbon dioxide reductase. ACS Catal 13:12374–12382. doi:10.1021/acscatal.3c02753

[35] Weghoff MC, Müller V. 2016. CO metabolism in the thermophilic acetogen Thermoanaerobacter kivui. Appl Environ Microbiol 82:2312–2319. doi:10.1128/AEM.00122-16 26850300 PMC4959504

[36] Jain S, Katsyv A, Basen M, Müller V. 2022. The monofunctional CO dehydrogenase CooS is essential for growth of Thermoanaerobacter kivui on carbon monoxide. Extremophiles 26:4. doi:10.1007/s00792-021-01251-y PMC8683389 34919167

[37] Schwarz FM, Ciurus S, Jain S, Baum C, Wiechmann A, Basen M, Müller V. 2020. Revealing formate production from carbon monoxide in wild type and mutants of Rnf- and ECH-containing acetogens, Acetobacterium woodii and Thermoanaerobacter kivui. Microb Biotechnol 13:2044–2056. doi:10.1111/1751-7915.13663 32959527 PMC7533326

[38] Zeldes B, Poehlein A, Jain S, Baum C, Daniel R, Müller V, Basen M. 2023. DNA uptake from a laboratory environment drives unexpected adaptation of a thermophile to a minor medium component. ISME Commun 3:2. doi:10.1038/s43705-022-00211-7 37938748 PMC9834392

[39] Lehmann M, Prohaska C, Zeldes B, Poehlein A, Daniel R, Basen M. 2023. Adaptive laboratory evolution of a thermophile towards a reduced growth temperature optimum. Front Microbiol 14:1265216. doi:10.3389/fmicb.2023.1265216 37901835 PMC10601643

[40] Kang S, Song Y, Jin S, Shin J, Bae J, Kim DR, Lee J-K, Kim SC, Cho S, Cho B-K. 2020. Adaptive laboratory evolution of Eubacterium limosum ATCC 8486 on carbon monoxide. Front Microbiol 11:402. doi:10.3389/fmicb.2020.00402 32218779 PMC7079680

[41] Jin H, Li X, Wang H, Cápiro NL, Li X, Löffler FE, Yan J, Yang Y. 2022. Anaerobic biohydrogenation of isoprene by Acetobacterium wieringae strain Y. mBio 13:e0208622. doi:10.1128/mbio.02086-22 36342171 PMC9765523

